# Long read sequencing reveals novel isoforms and spliceosome-mutant-enriched transcripts in AML and MDS

**DOI:** 10.64898/2026.05.20.726635

**Published:** 2026-05-27

**Authors:** Christopher A. Miller, Sridhar Nonavinkere Srivatsan, Michael H. Kramer, Sai Mukund Ramakrishnan, Catrina C. Fronick, Robert S. Fulton, Casey D. Katerndahl, Nichole M. Helton, Timothy J. Ley, Matthew J. Walter

**Affiliations:** 1 Department of Medicine, Washington University in St Louis, St Louis, MO, USA; 2 Siteman Cancer Center, St Louis, MO, USA; 3 Corresponding Author; 4 Lead contact

**Keywords:** transcript assembly, myeloid malignancies, acute myeloid leukemia, AML, myelodysplastic syndrome, MDS, alternative splicing, spliceosome, long read sequencing

## Abstract

The alternative splicing landscape of cancer transcriptomes remains poorly characterized, since short read sequencing cannot resolve complete transcript structures. Using the Oxford Nanopore cDNA platform, we generated nearly 2 billion long reads (median 25.8 million per sample) from 71 human samples, including 48 acute myeloid leukemia or myelodysplastic syndrome samples, 25 of which had splicing-factor gene mutations (in *SRSF2*, *U2AF1*, or *SF3B1*). An additional 23 samples were from sorted hematopoietic cell populations from healthy individuals. We identified 174,162 novel isoforms absent from the reference transcriptome, and proteomic validation confirmed that many are translated. We also identified isoforms enriched in spliceosome-mutant samples, and found proteomic evidence of frequent nonsense-mediated decay regulation of novel transcripts. This dataset is a valuable community resource, enabling detection of new transcripts in short read data sets. An interactive portal to explore splicing patterns in these data is available at https://leylab.org/isoforms/.

## INTRODUCTION

Surveys of cancer cell transcriptomes have been undertaken in nearly every solid tumor and hematologic malignancy^1^. These studies have predominantly employed short-read cDNA sequencing on the Illumina platform, which provides deep coverage of individual exons and splice junctions, but is limited in its ability to link these elements together into complete transcript structures^2^. More recently, long-read sequencing, on the Pacific Biosciences or Oxford Nanopore (ONT) platforms, has addressed this limitation, since they are capable of generating reads that span transcripts from end-to-end. Costs typically limit these experiments to lower read depths, ranging from 0.5 million reads to ~10M reads per sample^3-7^. These efforts have revealed previously unknown splicing patterns and transcripts, but thus far, each increase in long-read sequencing depth continues to reveal new transcripts, suggesting that current datasets capture only a small fraction of the overall transcriptional complexity present in cancer^8^.

This issue of transcriptional representation is especially salient in cancers with mutations in spliceosome component genes. Such mutations are frequently observed in myeloid malignancies such as myelodysplastic syndromes (MDS) and acute myeloid leukemia (AML). In AML, the most common splicing-factor mutation is *SRSF2*, found in around 7% of AMLs, while *U2AF1* and *SF3B1* are each mutated in about 3%^9^. These mutations are far more common in MDS patients, with as many as 50% harboring at least one mutation in a spliceosome gene^10-15^. Pan cancer studies also have shown that splicing genes are recurrently mutated in dozens of other tumor types, including lung, bladder, and endometrial carcinoma, with some recent studies suggesting that there may be more than 100 significantly mutated genes affecting splicing^16,17^.

The three genes assessed in this study directly impact either exon or splice-site recognition through distinct mechanisms, the net effect of which is to alter splicing of specific genes in ways that range from dramatic to subtle. Some are well-characterized: examples include “poison exon” creation, with premature termination codons that can be induced in *EZH2* (by *SRSF2* mutations)^18^ or *EIF4A* (by *U2AF1* S34F mutations)^19^, ultimately leading to nonsense-mediated decay and reductions in transcript and/or protein levels. Cryptic splice site usage, leading to reduction of *MAP3K7* levels (induced by *SF3B1* mutations)^20^, is another well-characterized event that contributes to tumor progression. These events do not occur in isolation though, and previous studies have described thousands of splice alterations induced by these mutations (some using long-read sequencing at lower depths in other cancers)^5,7,21-23^. Deciphering which of the transcriptional changes directly contribute to oncogenic phenotypes is an active area of investigation, but relies on the initial identification of high-quality full-length transcripts.

Despite the development of computational methods designed to detect these changes on a junction-by-junction basis using short read data, they are ultimately reliant upon having a comprehensive and representative transcriptome to guide alignments and abundance estimation. Previous long-read sequencing in AML and MDS suggests that existing transcriptome assemblies are incomplete, and that thousands of novel transcripts may exist. However, these studies were performed using lower sequence coverage depths (0.5-8 million reads per sample), and the higher-coverage cohorts have not included AMLs with splicing-factor mutations^4,22,24^. Given the lower depth and known ability of spliceosome mutations to create novel transcripts, these prior studies almost certainly have underestimated the total transcriptional diversity of AML.

In this study, we paired deeper long-read sequencing (median 25.8 million reads per sample) with a focus on splicing-factor mutations to search for transcriptional diversity that is absent from our current reference transcriptomes for AML and MDS. We also included sorted populations from healthy donor bone marrow samples, including CD34-positive progenitor cells, which may inform studies of clonal hematopoiesis and AML/MDS leukemogenesis, as well CD3 or CD19-positive lymphocytes, which may serve as references in studies of immune dysregulation, especially in a cancer context. Together, this approach reveals extensive and previously unrecognized isoform diversity in both myeloid malignancies and normal populations. By serving as a reference, this dataset will increase the power of the many existing short-read RNA sequencing studies, and, coupled with an easy-to-use web interface, will enable the community to undertake focused explorations of the role of transcriptome dysregulation in cancer.

## RESULTS

### Long-read cDNA sequencing from splicing-factor mutant AML and MDS

**Figure 1:**
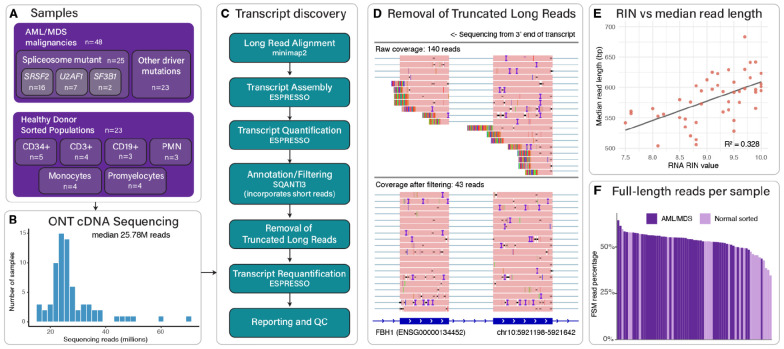
Cohort summary, assembly pipeline, and sequencing metrics (A) Overview of the samples used in this cohort (B) Number of ONT reads generated per sample (C) Overview of the transcript assembly pipeline - see Methods for details (D) Representative region in the *FBH1* gene illustrating many reads with 5’ truncation artifacts that were removed by filtering. (E) Samples with lower RIN values tend to have shorter overall reads, as measured by median read length (Pearson’s correlation coefficient p=2.11x10^−5^) (F) Percentage of non-truncated, full-splice match reads in each sample.

We assembled a cohort of 71 human subjects, which included 48 patients with either AML or MDS (Table 1, Figure 1A, Table S1). 25 of these were from cancers with mutations in spliceosome component genes. 16 cases had mutations at the common *SRSF2* hotspot (either P95[R/L/H], or G93GR), 5 had *U2AF1* mutations at position S34 (F/Y), 2 samples had *U2AF1* Q157P, and 2 samples had *SF3B1* mutations at the canonical K700E hotspot. As controls, we sequenced 23 samples from sorted hematopoietic cell populations from healthy individuals, including CD3+, CD19+, CD34+, promyelocytes (“Pros”), monocytes, and olymorphonuclear leukocytes (“PMNs”). All samples had libraries prepared using poly dT priming with the ONT cDNA PCB111 kit, then sequenced on a PromethION device, basecalled with Guppy, and aligned with minimap2, producing a median of 25.8 million reads per sample (range: 16.6 - 69.3), and 1.98 billion reads combined (Figure 1B, Table S1). In parallel, we sequenced each sample with the Illumina platform, using the Illumina Total RNA short-read kit, producing a median of 135.1 million 2x151 bp reads per sample. Due to the much longer read length, the median total basepairs sequenced per samples was 18.8 billion with the ONT long read platform, vs 12.2 billion with the Illumina short-read platform.

### A scalable pipeline for artifact-aware long-read transcript assembly

We explored several methods to assemble the nearly 2 billion reads^25,26^, but ultimately chose the ESPRESSO algorithm, in part because straightforward parallelization allowed us to construct a pipeline that could accommodate this quantity of reads (though some steps still required as much as 400GB of memory)^27^. Examination of the first-pass assembly of cDNAs revealed a substantial number of shortened isoforms, and examination of the underlying reads showed striking 3′ bias and 5′ truncation that appeared to be the primary source of these artifacts (Figure 1D). Previous publications have described these shortened reads^28^ and suggested a variety of contributing factors, including homopolymer tracts^29^ (an association we could not replicate, perhaps due to library preparation and basecalling improvements), reverse-transcriptase stalling^30^, or suboptimal flowcell performance - poor processivity of the pore enzyme or voltage spikes^31^. We observed a modest association between truncation hotspots and higher GC content (Figure S1), but not to a degree that could explain the majority of the shortened reads. We did, however, find that RNA Integrity Number (RIN) scores correlated with median ONT read length (Figure 1E), suggesting that choosing samples with high RNA quality is essential for maximizing read length and reducing truncations.

Most long read assemblers use similar approaches to handle ONT artifacts - including increased base substitution/indel error rates and homopolymer-associated errors that cause imprecise exon boundary alignment - but address read truncation differently^28^. Because ESPRESSO does not explicitly exclude truncated reads, we devised an iterative process to identify and exclude them (Figure 1C). Briefly, we assembled transcripts using the ESPRESSO algorithm, using its standard protocol, then annotated and filtered the resulting transcripts with SQANTI3^32^. Short-read data were provided to SQANTI3 to help refine splice junctions and inform levels of support for each transcript. SQANTI3’s built-in machine-learning classifier was used to designate transcripts as “Artifact” or “Isoform”, with the key discriminating variables including “bite” (signature of artifacts caused by RNA secondary-structure), read coverage, and the TSS-ratio, which compares short read to long read coverage at the 5′ end of transcripts to identify likely truncations. This approach removed 368,343 putative transcripts. Although this technique does not assign specific causes to each discarded transcript, manual review indicates that the majority were constructed from reads unlikely to be derived from genuine full-length cDNAs.

The corresponding truncated ONT reads were then removed, eliminating 47.2% of all reads (median per sample 53.8%, range 34.8 - 64.5%, Figure 1F). While some tools retain and flag these reads and use them to probabilistically estimate abundance, our focus is on isoform discovery, and since we had high depth data, we chose to remove them as part of a more stringent filtering strategy^26^. The proportion discarded in cryopreserved tumor samples was higher than that of fresh flow-sorted healthy normal samples (median 54.5% vs 49.5%, Wilcoxon p=0.002). The remaining reads were re-quantified with ESPRESSO and then filtered to require novel isoforms to be called in at least two samples and supported by 5 total long reads - thresholds that prioritized high-confidence results over sensitivity. Long transcripts were substantially underrepresented in the long read assembly, due to poor coverage of the 5′ ends. We therefore retained all Ensembl transcripts, regardless of long-read coverage, reducing false negatives while focusing our discovery efforts on novel transcripts, especially those caused by aberrant splicing^33^.

### Novel transcripts expand the reference transcriptome

**Figure 2:**
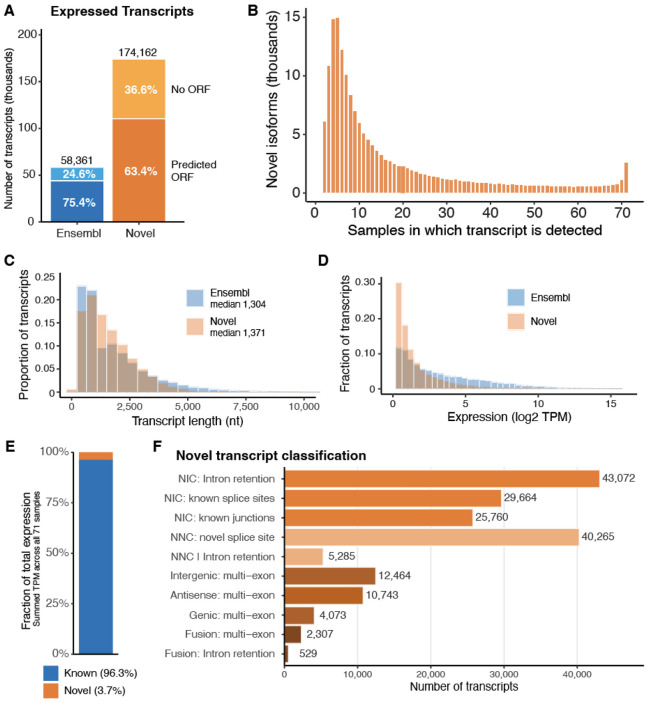
Novel Isoforms discovered in this dataset (A) Transcripts with measurable expression in this cohort (only 58,361 of 206,601 Ensembl transcripts were detected in these tissues). Significantly fewer novel transcripts are predicted to have open reading frames. (Fisher’s exact test, *p*<2x10^−16^). (B) The number of samples expressing each novel transcript (C) Distribution of transcript lengths, comparing known Ensembl to newly identified transcripts (D) Distribution of total expression levels (TPM) for known and novel transcripts, summed across all samples (E) Fraction of total expression that comes from known vs new transcripts (F) Classification of novel transcripts from SQANTI3. (NIC = Novel In Catalog, NNC = Novel Not in Catalog)

Despite this conservative approach, the resulting hematologic transcriptome assembly contained 174,162 novel transcripts, along with 206,601 known Ensembl transcripts (Figure 2A). Of these novel transcripts, 51.2% were identified in at least 10 samples, with 1.5% detected in all samples we examined (Figure 2B). These new transcripts satisfied multiple criteria for plausibility, including lengths comparable to known Ensembl transcripts (Figure 2C). A proportionately smaller fraction of new transcripts exceeded 3,000 nucleotides in length, reflecting the 3′ bias in this long read data (known: 17.9%, novel: 11.0%, Chi-squared *p* < 2x10^−16^). Novel transcripts exhibited lower overall expression (Figure 2D) and collectively represent 3.7% of the total mRNA quantified in these cells (Figure 2E). This lower abundance matched our expectation that more highly expressed isoforms would historically be easier to detect. Nonetheless, many are expressed at appreciable levels with 10,186 transcripts that contribute over 10% of their corresponding gene’s expression. Together with extensive manual review of putative transcripts, these characteristics give us high confidence that the transcripts reported here are predominantly genuine isoforms thave have not been described in the current Ensembl reference transcriptome.

Intron retention represents the most common class of novel transcript (Figure 2F). Novel splice sites and combinations of known splice sites and junctions make up a plurality of the rest - these categories include traditional short-read classifications such as exon skipping or alternate 3’ or 5’ splice sites. 23,208 of these transcripts (13.3%) were classified by SQANTI as coming from 7,048 novel genes, where the transcript coordinates map entirely outside known annotations. These arise from a variety of sources, and 992 intersect with known long non-coding RNAs^34^. Another small fraction represent partial overlaps to reported sequences not represented in Ensembl - predicted genes, other small RNA species, or antisense transcripts - but the majority represent expression from sequences that are not well annotated.

### Proteomic evidence for translation of novel transcripts

**Figure 3:**
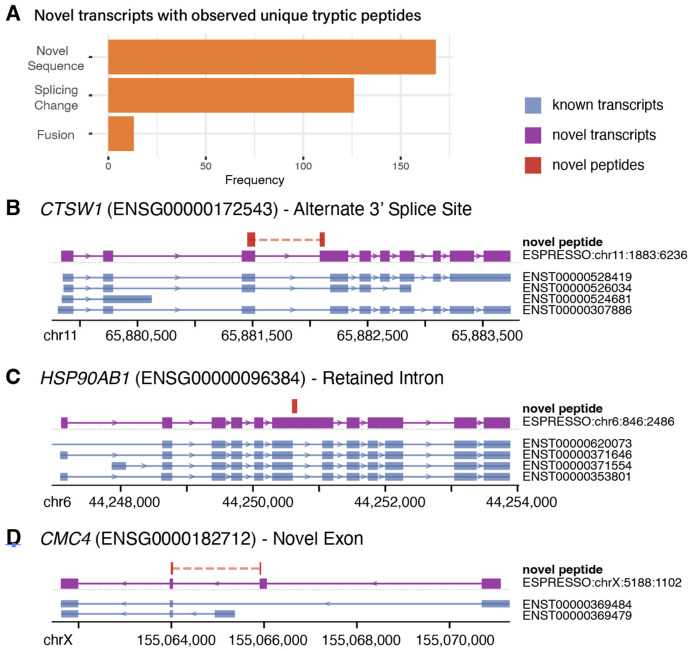
Proteomic validation of novel transcript translation (A) Classification of novel peptides detected in any of 24 AML samples evaluated with TMT proteomics (B) Schematic of the *CTSW* gene, showing known transcripts, novel transcripts, and the location of the translated sequence that gives rise to the novel peptide, in this case from sequence spanning the junction between a known exon and new sequence created from alternate 3’ splice site usage (C) schematic of the *HSP90AB1* gene, showing a novel peptide translated from retained intron sequence (D) schematic of *CMC4*, which has a novel peptide created from sequence that spans a splice junction from a known exon into a novel exon.

24 of the AML samples in this study had previously-generated deep-scale proteomic data, giving us the ability to search for evidence of novel isoform translation^35^. Several factors make such identification challenging: 1) Mass spectrometry has inherently low sensitivity, and despite generating a modern “deep” proteome, the Kramer et al. study detected a mean of only 8,976.2 proteins per sample (range 8,698-9,290) with the Tandem Mass Tag (TMT) approach 2) Most novel isoforms are expressed at relatively low levels, implying that their protein abundance may be low as well. 3) Only a subset of peptides ionize and transmit efficiently enough to be detected by mass spectrometry, and 4) Any given event (e.g. novel exon or junction) may not generate a tryptic peptide detectable by mass spectrometry workflows. Despite these limitations, we identified 307 distinct high-confidence peptides from 273 different transcripts and 261 genes (Figure 3A, Table S2). These fall into several categories, with 160 (52.1%) representing new mRNA sequence derived from known genes -- generated by intron retention, alternative 3′ or 5′ splice sites, frameshifts, or use of new exons. Another 126 peptides (41.0%) were derived from splicing alterations (e.g. exon skipping) that create fusion peptides absent in the reference proteome. 8 (2.6%) originated from novel protein coding sequences that do not exist in the reference, and 13 (4.2%) were from fusion events, which were primarily read-through transcripts from neighboring genes. Figures 3B-D provide representative examples of novel peptides arising from alternate 3′ splice-site usage, a retained intron event, and a new exon not present in the reference transcriptome. Although many of these may generate neoantigens, that could theoretically be recognized by the immune system, the low overall expression of these transcripts and peptides limits their attractiveness as therapeutic targets.

Standard protein database searches often use the “reviewed” FASTA references from UniProt, which provide a less comprehensive set of protein sequences than those described in either Ensembl or our expanded assembly^36^. When using predicted protein-coding sequences from our extended transcriptome as the basis for a search, we identified thousands of additional peptide matches. These largely fell into two categories: 1) fully tryptic peptides mapping to known Ensembl transcripts, but absent from the UniProt database or 2) semi-tryptic partial matches that could be produced by alternative splicing. Specifically, when novel transcripts contain premature termination codons, the resulting translated peptide would match the reference, but terminate early, before the expected tryptic cleavage site. Similarly, new transcription start sites may generate peptides with apparent N-terminal truncation. Since we could not rule out protein degradation or non-specific proteolytic cleavage as a cause of these observations, we did not treat them as being strong evidence for novel isoform translation. Collectively, these analyses suggest that more comprehensive reference databases may benefit proteomics studies and also provide evidence that many novel transcripts we identified are valid and being translated into proteins.

### Characterization of nonsense-mediated decay in novel transcripts

**Figure 4:**
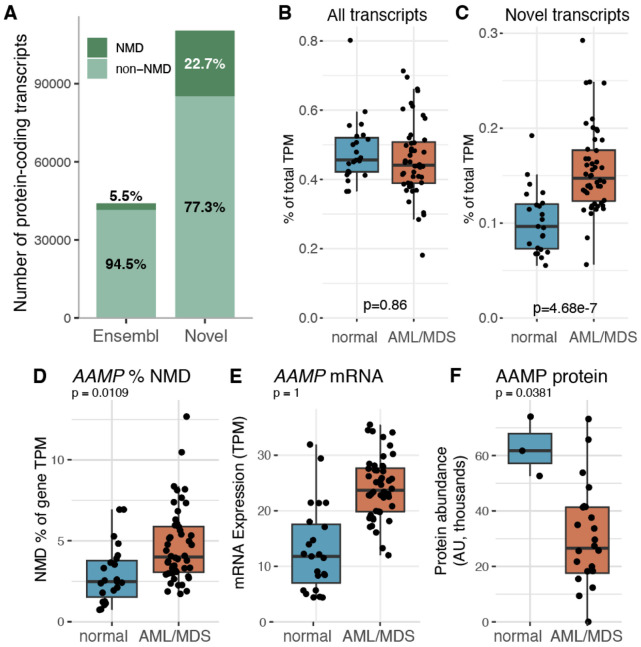
Nonsense-mediated decay in novel transcripts (A) Number and percentage of predicted protein-coding transcripts predicted to undergo nonsense-mediated decay (NMD) (Fisher’s exact test: *p*<2.2x10^−16^). (B) Percentage of all mRNA expression that comes from NMD transcripts in normal healthy cells (blue) and AML/MDS tumor cells (red) (Wilcoxon rank sum *p*=0.86). (C) Percentage of all mRNA expression that comes from novel NMD transcripts in normal healthy cells (blue) and AML/MDS tumor cells (red) (Wilcoxon rank sum, *p*=4.68x10^−7^) (D) Increased percentage of overall *AAMP* transcripts coming from NMD transcripts in tumors as compared to normal cell populations (Wilcoxon rank sum, *p* = 0.0109) (E) mRNA expression of *AAMP* in tumors compared to normal healthy cells (F) Protein abundance of AAMP in tumors compared to normal cell populations (Wilcoxon rank sum, *p*=0.0381) All *p* values BH-adjusted.

The majority (63.4%) of novel transcripts are predicted to have protein-coding open reading frames (ORFs), but these also show significantly higher levels of predicted nonsense-mediated decay than known transcripts (*p* < 2.2x10^−16^, Figure 4A), a finding consistent with previous reports^37^. While tumors and healthy cells have similar overall levels of NMD transcript expression, a larger proportion of this expression comes from novel isoforms in tumor samples (Figure 4B,C, *p*=4.68x10^−7^), suggesting that the contribution of NMD may have previously been underestimated in tumor cells. Overall, 417 genes had significantly higher levels of NMD transcript accumulation in tumor samples than healthy cells, and 337 genes had higher levels specifically in splicing factor-mutant tumor samples (Table S3, Table S4). Both of these NMD-enriched lists had overrepresentation of genes in RNA metabolism and mRNA splicing (largely driven by novel transcripts) supporting the idea that NMD is a common mode of self-regulation for these pathways and a common dysregulated feature in cancer^38-41^.

NMD-predicted transcripts are regulated at multiple levels. Some NMD transcripts are quickly degraded, reducing total mRNA abundance, while other transcripts are stable, with regulation occurring via altered translation or protein degradation^42^. By integrating the proteomics data above with our expanded transcriptome, we sought to identify AML-relevant genes subject to NMD-mediated regulation that would be invisible without both data layers. We specifically looked for genes where: 1) we observed no significant reduction in gene-level mRNA expression levels in tumors, 2) NMD transcripts made up an increased proportion of the gene’s expression, and 3) protein abundance was significantly lower in tumor samples (all using Wilcoxon rank sum test, BH-corrected *p*<0.1). We identified 70 such genes, including *AAMP* (Figure 4D-F). In *AAMP*, this NMD isoform shift occurs primarily within novel transcripts. Across tumor samples, a mean of 63% of NMD transcripts were novel (range 37-80%). The large number of newly detectable NMD transcripts in this assembly will facilitate more detailed and accurate characterization of these regulatory effects in future studies, especially when coupled with proteomics.

### Healthy donor cells harbor population-specific novel transcripts

**Figure 5:**
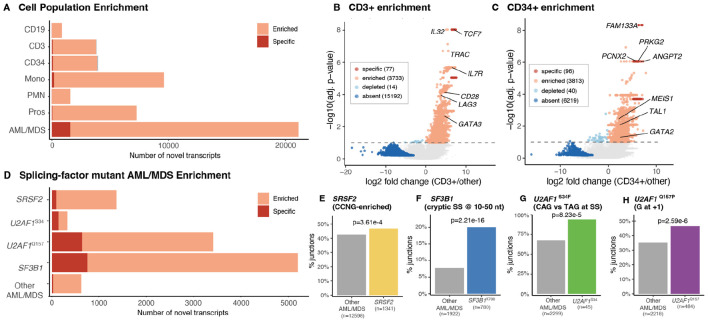
Novel transcript enrichment in sample subsets (A) Quantification of novel transcripts that are enriched in (pink) or specific to (red) sorted populations or tumor cells as compared to all other samples combined (B) novel transcript enrichment in CD3+ sorted T cells (C) novel transcript enrichment in CD34+ sorted stem/progenitor cells (D) Counts of novel transcripts that are enriched (pink) or specific to (red) splicing-factor mutant AML/MDS samples, as compared to all other AML/MDS and CD34+ stem/progenitor cells. (E) Enrichment of CCNG motifs (vs GGNG) in novel transcripts from *SRSF2*-mutant samples (Wilcoxon rank sum *p*=3.61x10^−4^) (F) Fraction of alternative 3’ splice site junctions that occur in the expected interval (10-50bp upstream of a known acceptor site) in SF3B1 samples (Fisher’s exact test *p*=2.21x10^−16^) (G) Fraction of alternative 3’ splice site junctions that match the CAG motif vs TAG motif in samples with *U2AF1*^S34F^ mutations (Fisher’s exact test *p*=8.23x10^−5^). (H) Fraction alternative 3’ splice site junctions that have G at position −1 vs C in samples with *U2AF1*^Q157P^ mutations (Fisher’s exact test *p*=2.59x10^−6^)

Since we generated data from sorted populations of bone marrow cells from healthy donors, we sought to identify novel isoforms that were restricted to specific hematopoietic lineages and/or differentiation states. Each population was compared against all other samples, identifying isoforms that were either specific to that population (i.e. absent in all others) or statistically enriched in that population (Figure 5A-B, Figure S2, Table S5). Each sorted cell type contained high numbers of such events, ranging from 850 in CD19-positive cells to 9,580 in Monocytes. AML/MDS cells harbored more specific/enriched novel transcripts (21,101), perhaps due to their altered biology and the greater statistical power that comes from a larger number of samples. Many of these population-enriched novel transcripts occurred in well-known cell lineage regulated genes, like *TCF7* in CD3-positive T cells (Figure 5B), or *FAM133A* in CD34-positive myeloid progenitor cells (Figure 5C), revealing new splicing patterns in these genes. Overrepresentation analysis confirms that genes bearing these novel transcripts are significantly associated with known lineage-relevant pathways, like “T Cell Activation” and “Lymphocyte Differentiation” for CD3+ cells. (Table S6).

### Spliceosome mutations are associated with characteristic signatures in novel transcripts

The splicing-factor gene mutations in our cohort are all known to cause specific, well-defined alterations in splice site recognition and usage. We therefore hypothesized that novel transcripts which are enriched in splicing-factor-mutant tumors would bear the sequence signatures characteristic of the respective mutant splicing factor. We performed the same enrichment analysis described above on the AML/MDS cohort, stratifying samples by the presence of mutations in *SRSF2*, *SF3B1*, and *U2AF1* (comparing mutations at amino acid S34 vs. Q157, since they are known to have distinct functional effects^43^, Figure 5D, Figure S3, Table S7) We then compared these samples to non-splicing factor mutated AML/MDS samples to determine the proportion of the novel splicing events that contained the canonical splice site sequence motif associated with each mutation.

Novel transcripts enriched in *SRSF2*-mutant samples displayed the expected shift towards CCNG over GGNG motifs in exon splicing enhancers (*p*<2.1x10^−5^)^18,44^. Similarly, novel transcripts enriched in *SF3B1*-mutated samples showed preferential use of cryptic 3’ splice sites located 10-50 nucleotides upstream of a canonical acceptor site (*p*=2.7x10^−19^)^45^. In *U2AF1* S34F-mutant samples, cytosine at the −3 position, preceding the splice-acceptor AG dinucleotide, was significantly enriched relative to the wild-type thymine preference in novel transcripts (*p*=0.001). *U2AF1* Q157P mutant samples showed evidence of increased guanine residues at the +1 position relative to the 3’ splice site (*p*=2.1x10^−4^, Figure 3b)^46^. Overrepresentation analysis of novel transcript-harboring genes with our four splicing-factor gene mutations shows significant enrichment in pathways like “RNA splicing” and “mRNA processing” (Table S8), which support the fact that these factors are known to function in self-regulatory feedback loops^47,48^. *DNMT3A* inactivation in mouse hematopoietic cells has been recently postulated to cause altered mRNA splicing, but data from the 7 *DNMT3A*-mutated samples in this study did not show any evidence that would support the proposed mechanism^49^. Collectively, these results provide evidence that mutationally-driven splicing mechanisms contribute to the generation of novel transcripts in AML/MDS.

### Detecting YBX1 loss-of-function splice variants in SRSF2-mutant samples

We applied rMATs-long to these data, to identify differentially abundant transcripts between sample groups, as defined above. We identified 1,524 transcripts that were differentially expressed between tumors and healthy donor derived hematopoietic cells, and between 755 and 2,642 differentially expressed in comparisons between specific population or tumor subsets (Table S9). Within these groups, 24% of differential isoforms were from novel transcripts. Among the most notable findings was a novel isoform of the *YBX1* gene, a gene with established roles in AML pathogenesis^50-53^. This transcript (ESPRESSO:chr1:948:464) is nearly absent in most AML/MDS samples and healthy donor derived hematopoietic populations but represents up to 25% of YBX1 transcripts in *SRSF2*-mutant samples. It also has less pronounced overexpression in *U2AF1*-mutant, but not *SF3B1*-mutant, malignancies. This exon skipping event causes loss of much of the functional cold-shock domain, with predicted deleterious effects. Further work will be needed to understand the functional consequences of this isoform switch and whether it contributes to AML/MDS pathogenesis.

### A more complete transcriptome assembly enables new insights from short-read data

We next evaluated whether the expanded transcriptome could improve transcript quantification from short read RNA-seq data. We first realigned and requantified the short-read RNAseq data from 65 of the same samples used to construct this assembly. Importantly, these short-read sequences were not used to create this assembly directly - their sole prior use was as a filter to exclude novel long read transcripts with insufficient short read support. Reprocessing with the expanded new transcriptome enabled detection of 95.2% (165,724/174,162) of the novel transcripts, none of which could be reported from the original processing of the short-read data. More nuanced splice-junction analyses would likely recover some of these events, but using a more comprehensive transcriptome means that they are immediately included in the primary results.

To further evaluate the utility of this expanded transcriptome reference, we quantified transcript abundance in 130 additional short read RNAseq samples, aligning reads and quantifying with this expanded transcriptome. In total, 96.2% of novel transcripts (167,679/174,162) were detected across this extension cohort (Figure 7A). The alternative *YBX1* transcript described above (ESPRESSO:chr1:948:464) was detected at high levels (>5 CPM) in 6 of those samples. When cross-referenced with DNA sequencing data, 5 of the 6 AMLs harbored *SRSF2* P95 mutations, and the other contained a *U2AF1* Q157P mutation, further validating the mutational association described above. Notably, *YBX1* had no differential expression at the gene level in these samples, so *YBX1* and its transcripts would never have been flagged as a gene of interest by such analyses. Finally, we aligned single-cell RNAseq (5′ data kit from 10x Genomics) from 6 AML samples (not from this cohort) to this expanded transcriptome reference. Since sensitivity is fundamentally low and genes have limited internal coverage, scRNA pipelines typically aggregate to the gene level. Thus, we focused only on transcripts from novel genes, detecting 15,084 of 27,754 (54.3%) novel genes in the 6 samples. 27,093 out of 27,754 cells (97.6%) contained evidence of at least one novel gene. These results underscore that many transcripts are inaccessible using the standard Ensembl transcript reference, but are recoverable using this expanded reference transcriptome, even when using short-read data.

### A resource for improving transcriptomics in blood cancers

Along with this expanded transcriptome, we developed a “Transcriptome Explorer” web portal that provides detailed information on transcripts identified in this study, specific splicing patterns, and gene and transcript abundance (Figure 7B/C). The portal was built on top of the IsoVis software, heavily modified and expanded to add functionality specific to this data set^54^. It is freely available for use at https://leylab.org/isoforms/. The source code can be found at https://github.com/chrisamiller/aml-transcriptome, and the version referenced in this publication is archived at https://doi.org/10.5281/zenodo.20314851. The portal also hosts key datasets and a GTF ready to be dropped into RNAseq pipelines.

## DISCUSSION

In this study, we deeply sequenced long read RNA from a broad set of bone marrow samples from healthy donors, or patients with myeloid malignancies, and then created a transcript assembly that is more comprehensive than the Ensembl reference transcriptome. The resulting catalog -- 174,162 novel transcripts alongside 206,601 known Ensembl isoforms -- adds approximately 1.5-fold more novel transcripts than reported in a previous, lower sequencing depth study of AML (direct comparison to the work of Shi, et al., was not possible because their assembly is not public)^24^. This expansion can be primarily attributed to two factors: The sequencing depth we achieved (nearly 2 billion reads, median 25.8 million per sample), and the inclusion of 25 splicing-factor mutant samples, which are a particularly rich source of transcriptome diversity that has not been explored with this depth in AML/MDS samples.

Proteomics data contributed greatly to this study, and pairing it with this kind of deep transcriptional profiling suggests a paradigm for future studies. Detection of 307 high-confidence novel peptides demonstrates that some novel isoforms are indeed translated, and that larger transcript assemblies can expand the reach of proteomics studies, by recovering biologically-relevant peptides that are currently ignored. We also identify genes like *AAMP* where there is no reduction in overall mRNA abundance, but protein levels are reduced. When coupled with observed isoform shifts towards NMD-sensitive transcripts, it suggests that NMD regulation is occurring, but that it is invisible to gene-level analysis. These findings emphasize that comprehensive transcript catalogs are a prerequisite for accurately characterizing the scope of NMD regulation in cancer cells.

The inclusion of flow-sorted bone marrow subpopulations allowed us to deeply characterize cell types that may only make up a few percent of a typical bone marrow sample, but play an outsized role in cancer. These included CD3+ T cells and CD19+ B cells relevant to immune dysregulation and CD34+ stem/progenitor cells which are often the cell of origin for MDS and AML. Each population contains hundreds to thousands of population-enriched novel transcripts, often occurring in lineage-relevant genes. These data may be useful for studies of immune function in many tumor types.

Prior work in splicing-factor gene mutant AML/MDS has characterized disruptions to splicing primarily at the level of individual junctions. This full length isoform assembly places aberrant junctions into the context of the entire transcript structure. This work also showed that although we can detect the mutation-specific signatures of splicing dysregulation, most novel transcripts in splicing-factor mutant samples are not specific to a given genotype: fewer than 0.1% of novel transcripts were exclusively found in such samples. Instead, these data support a model in which spliceosome mutations shift the relative ratios of canonical splice-sites, enriching for low-abundance isoforms, rather than creating entirely new ones^45,55^. This more comprehensive catalog of altered splicing in these samples will aid in detailed studies of specific genes and pathways that may be driving oncogenesis in these cancers.

Another illustration of the value of this resource is the identification of a novel *YBX1* exon-skipping isoform, enriched in *SRSF2*-mutant, and to a lesser extent, *U2AF1*-mutant AML and MDS. This isoform is nearly absent in other AMLs, and in healthy donor cells, but represents up to 25% of *YBX1* transcripts in SRSF2-mutated samples. We provide validation of the association using 130 short read AML samples, showing that the samples with high abundance of this isoform have *SRSF2* and *U2AF1* mutations (including one cryptic *SRSF2* mutation that was previously missed). YBX1 is one of several strong candidates for functional follow-up studies, and represents a broader class of spliceosome-driven isoform switches that do not alter overall gene expression, but can be readily detected at the transcript level by using a more comprehensive reference assembly.

This expanded transcriptome resource is immediately applicable to existing RNA-seq datasets, without having to generate new data. When reanalyzing short-read RNAseq samples, both from these samples and a extension cohort, we were able to detect most of the novel isoforms. Further, in a reanalysis of 6 single-cell RNAseq samples from AML patients, we detected more than 50% of the novel genes identified in our new reference transcriptome. Applying these same techniques to other large short-read cohorts, like Beat AML or TCGA AML, represents an immediate opportunity to look for previously overlooked isoform shifts that may be relevant for disease pathogenesis^56,57^.

This study represents the deepest long read transcriptome of AML/MDS to date, and reveals new and more extensive isoform diversity than previously described. The expanded transcript assembly is immediately applicable to existing and future studies of these diseases, including short read, single cell, and proteomic datasets. The “Transcriptome Explorer” web portal is designed to make these data accessible, even to those who are not bioinformatics-savvy, and will enable the broader community to pursue transcript-level hypotheses in their genes and pathways of interest. Studies built upon this reference will be well-positioned to connect specific isoform shifts to clinical phenotypes, therapeutic approaches, and the mechanisms leading to spliceosome-driven oncogenesis.

### Limitations of the study

To maintain compatibility with other AML datasets, this assembly is based on version 95 of Ensembl, rather than the latest release (v115). A post-hoc analysis found that 12.3% of our novel transcripts are now present in Ensembl 115. This reflects the increasing breadth of databases, as studies like this one become available, and serves as further validation of the quality of this assembly.

3’ bias of the long read data represents perhaps the most serious limitation of this study. ONT sequencing with poly-A+ priming leads to limited representation of novel transcripts from longer genes, as illustrated by the near-complete absence of full-length reads from either *DNMT3A* (canonical transcript length: 4,279 basepairs) or *NF1* (12,373 bp) across this cohort. Direct RNA sequencing might circumvent some of this bias, but current yields are low, and further innovation will need to occur before it is viable for producing deep transcriptomes of the type we present here. Alternative platforms, such as Pacific Biosciences, offer comparable long reads, but the resulting datasets suffer from many of the same biases. Overall, our data suggests that almost half of all reads produced from these samples do not represent full-length transcripts, so we acted conservatively, discarding them to prioritize confident assembly, at some cost to sensitivity. Some factors are within user control, however, and the correlation that we observe between RIN and sequence length suggests that RNA integrity is a meaningful predictor of long read sequence length and quality. We also found that flow-sorting may compromise RNA quality, perhaps even more so than the freeze-thaw cycle involved in processing AML/MDS cryopreserved samples. These are all findings with practical implications for cohort design.

Additional limitations include the small number of samples from *SF3B1* mutant (n=2) and *U2AF1* Q157P mutant (n=2) samples, which limit statistical power, and the restricted set of sorted cell populations from healthy donor bone marrows. Future studies may fill the gaps by expanding the repertoire of cell-types, as well as exploring samples with mutations in other spliceosome components, like *ZRSR2*. In addition, proteomics sensitivity remains a challenge, and we anticipate that deeper proteomic coverage would also identify a substantially larger number of peptides derived from novel isoforms.

## STAR★METHODS

### Sequence generation

The quality of the total RNA was determined by the Agilent Bioanalyzer (RIN ranged from 6.7-10), the quantity was determined by the Qubit Flex (HS RNA kit), and the purity was assessed on the nanodrop (expected values 260/280 ~2 and 260/230 ~2-2.2). 200ng of total RNA was input into the PCR-cDNA Barcoding Kit (Oxford Nanopore, SQK-PCB111.24). The reverse-transcribed (RT) sample was split into two second strand PCR reactions (2x50uL reactions with 5uL of RT product in each reaction per the manufacturer’s protocol). 14 cycles of PCR with 8-minute extensions were performed. Unique barcoded adaptors were ligated per sample. The barcoded libraries were pooled in equal molar ratios and loaded across multiple flow cells to mitigate flow cell to flow cell pore count variability. ~50fmol of pooled library was loaded per PromethION flow cell (R9.4.1, Oxford Nanopore, FLO-PRO002) targeting 30-40M reads per sample. Sequence data was base called on-instrument using the Super-accurate basecalling model (Guppy 6.4.6). Data was collected for ~90 hours generating >140M reads per flow cell.

### Data Processing

Reads were trimmed with pychopper (https://github.com/epi2me-labs/pychopper), then aligned to human genome GRCh38 with the chromosome 21 U2AF1 patch^64^ using minimap2 with parameters `-ax splice -uf`^65^. Quality control statistics were generated using NanoPlot version 1.41.0^59^.

### Transcript Assembly and filtering

We first ran the standard ESPRESSO workflow on the aligned reads, using version 1.3.2, parallelized per-chromosome in order to facilitate lower memory usage and increased speed^27^. Ensembl version 95 transcripts were provided as a baseline reference^33^. Annotation of the assembled transcripts was performed with SQANTI3 version 5.2.1^32^, providing the short-read RNAseq to help validate long-read transcript models. SQANTI3 was then used to filter the data, using the built-in machine learning approach with default parameters. Novel transcripts that passed these filters were merged with the original Ensembl transcripts. ESPRESSO quantification was then re-run so that reads linked to removed transcripts had a chance to be reassigned. The read assignments from this step were then processed and all reads not categorized as full-splice-match are removed as probable truncation artifacts. Quantification was run a third time on these full-splice-match reads to produce a final long-read expression matrix. Coverage filters were then applied, to remove any transcript not present in at least two samples and not supported by at least 5 reads. Comparisons of this assembly to other ensembl versions or to LNCipedia were carried out using gffcompare 0.12.6.

### Proteomics

Proteomic data was generated and processed as described previously^35^, and abundance values from that publication were used in all plots, unless otherwise specified. For detection of novel peptides, open reading frame predictions from SQANTI3 were used to identify peptides unique to novel transcripts. FragPipe version 22.0 was used to re-search the TMT data against this set of novel protein sequences using the default TMT-11 workflow parameters (using trypsin digest and allowing 2 missed cleavages)^61^. The resulting protein abundance files were compared to all peptides from Ensembl v95 to remove known sequences. Each peptide was then classified according to its mechanism of creation (splicing, fusion, frameshift, etc).

### Transcript Enrichment and NMD

For population-based enrichment, each sorted normal population was grouped individually with tumors combined as a separate group. Transcripts were tested for enrichment/depletion in a particular population using a two-sided Wilcoxon rank-sum test. The “specific” label was applied when a transcript was only found in one group, regardless of significance. The “absent” label similarly required zero presence in a particular group. For splicing-factor mutant enrichment, the same statistics were applied, but the samples were grouped into four splicing groups (SRSF2, U2AF1_S34F, U2AF1_Q157P, and SF3B1), along with all other tumors and CD34-positive cells as comparators. Nonsense-mediated decay predictions were extracted from SQANTI3 annotations.

Differentially expressed isoforms were detected using rMATS-long v2.0.1, with parameters `--delta-proportion 0.1` and `--adj-pvalue 0.1`, which also requires average reads per group > 10.0 and transcript CPM >= 5% of gene CPM.

### Data Portal

The data exploration portal was based on the IsoVis software, with substantial modifications made to enable abundance plotting, optimization to allow for larger datasets, exposing additional transcript information, and providing accessory scripts for preparing data.^54^. The resulting code is freely available at https://github.com/chrisamiller/aml-transcriptome, and the version described in this manuscript has been stably deposited at https://doi.org/10.5281/zenodo.20314851.

## Supplementary Material

Supplement 1 Table S1: Detailed characteristics of the patient cohort and ONT sequencing, related to Figure 1

Supplement 2 Table S2: High-confidence peptides detected from novel transcripts, related to Figure 3

Supplement 3 Table S3: Genes with higher levels of NMD transcript accumulation in tumor samples, related to Figure 4

Supplement 4 Table S4: Genes with higher levels of NMD transcript accumulation in splicing-factor mutant tumor samples, related to Figure 5

Supplement 5 Table S5: Novel transcripts specific to or enriched in a given population, related to Figure 5

Supplement 6 Table S6: Overrepresentation analysis of genes harboring enriched novel transcripts in specific populations, related to Figure 5

Supplement 7 Table S7: Novel transcripts specific to or enriched in categories of splicing-factor-mutant tumors, related to Figure 5

Supplement 8 Table S8: Overrepresentation analysis of genes harboring enriched novel transcripts in splicing-factor mutant tumor samples, related to Figure 5

**Figure 6:**
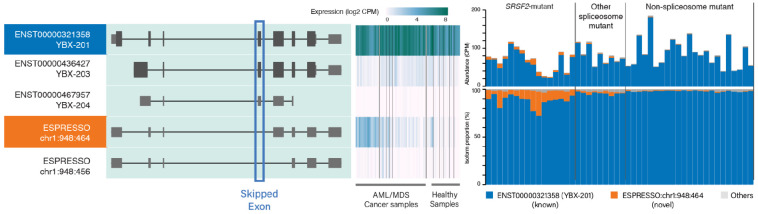
A novel transcript of YBX1 (orange) caused by exon skipping (left), that has increased abundance in *SRSF2*-mutant and *U2AF1*-mutant AML/MDS samples (center and right).

**Figure 7:**
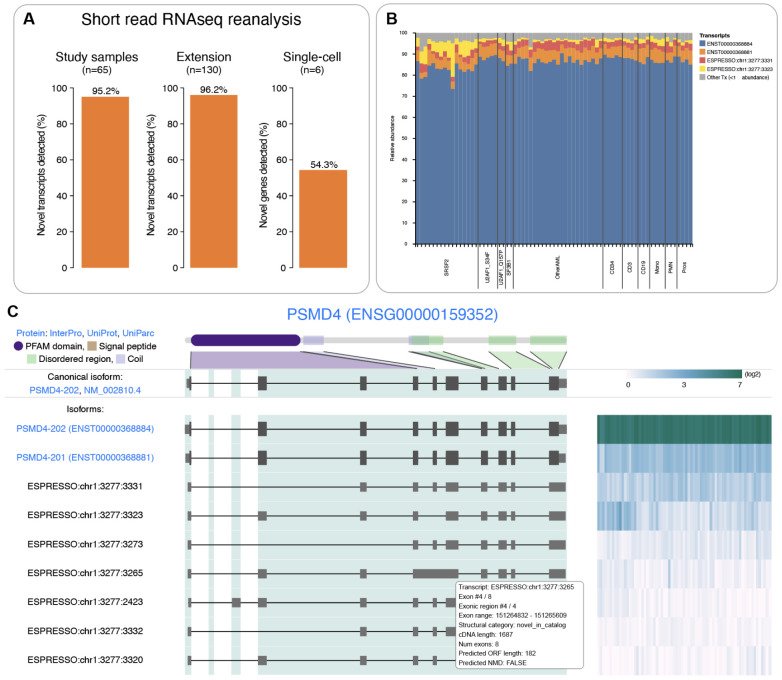
A resource for exploring the expanded transcriptome (A) Processing short reads with the expanded transcriptome enables primary detection of most novel isoforms, both in short-read data from this cohort (left) an extension short-read cohort (center). Novel genes are similarly newly accessible in single-cell RNAseq data (right). (B) Screenshot of transcript abundance generated from the web portal (C) Screenshot of the web portal displaying transcript structures (left), abundance heatmaps (right), and detailed transcript information (popup). Additional visualization layers and filters are available through the portal’s menus.

Supplement 9 Table S9: Differentially expressed isoforms between specific population or tumor subsets from rMATS-long, related to Figure 6

Supplement 10 Document S1: Figures S1-S3

## Figures and Tables

**Table 1:** Summary statistics of patients in the cohort

**Age (mean +/− SD)**	63.5 +/− 17.9
**Sex (n, %)**	
Male	33 (68.7%)
Female	15 (31.2%)
**Diagnosis (n, %)**	
MDS	3 (6.25%)
Primary AML	42 (87.5%)
Secondary AML	3 (6.25%)
	
**Key Mutations (n, %)**	
*SRSF2*	16 (33.3%)
*U2AF1*	7 (14.5%)
*SF3B1*	2 (4.2%)
*DNMT3A*	7 (14.5%)
*FLT3*	4 (8.3%)
*RUNX1*	2 (4.2%)
*TP53*	1 (2.08%)
	
**FAB Types**	
M0	5 (10.4%)
M1	14 (29.2%)
M2	12 (25%)
M3	1 (2.1%)
M4	6 (12.5%)
M5	3 (6.2%)
unknown/NA	7 (14.5)

**Table:** KEY RESOURCES TABLE

REAGENT OR RESOURCE	SOURCE	IDENTIFIER
**Critical commercial assays**		
PCR cDNA sequencing kit	Oxford Nanopore Technologies	SQK-PCS111
PromethION flow cell (R9.4.1)	Oxford Nanopore Technologies	FLO-PRO002
TruSeq Stranded Total RNA Gold kit	Illumina	RS-122-2303
		
**Deposited data**		
Long read sequence data from 71 samples	This publication	dbGaP study phs000159
Short read sequence from 201 samples	This publication	dbGaP study phs000159
Reference genome GRCh38	Genome Reference Consortium	https://www.ncbi.nlm.nih.gov/datasets/genome/GCF_000001405.40/
Chr21 U2AF1 patch for reference genome	NCBI	https://ftp.ncbi.nlm.nih.gov/genomes/all/GCA/000/001/405/GCA_000001405.15_GRCh38/seqs_for_alignment_pipelines.ucsc_ids/GCA_000001405.15_GRCh38_GRC_exclusions.bed
Reference Transcriptome - Ensembl v95	Ensembl^33^	https://ftp.ensembl.org/pub/release-95/gtf/homo_sapiens/Homo_sapiens.GRCh38.95.gtf.gz
LNCipedia v5.2	Volders, et al. 2019^34^	https://lncipedia.org/download
Human AML proteomics data	Kramer, et al 2022^35^	https://storage.googleapis.com/tcga_shiny/TCGA_Proteomics_app/Supplemental%20Table%203.xlsx
Reference Proteome	Uniprot^36^	https://rest.uniprot.org/uniprotkb/stream?compressed=true&format=fasta&query=%28*%29+AND+%28model_organism%3A9606%29+AND+%28reviewed%3Atrue%29
**Software and algorithms**		
Guppy v6.4.6	Oxford Nanopore	https://nanoporetech.com/software/other/guppy
pychopper v1.41.0	Oxford Nanopore	https://github.com/epi2me-labs/pychopper
minimap2 v2.17	Li, H. 2018^58^	https://github.com/lh3/minimap2
NanoPlot	De Coster, W. 2023 ^59^	https://github.com/wdecoster/nanoplot
R v4.3.3	R Development Core Team, 2024^60^	https://www.R-project.org/
FragPipe version 22.0	Kong, et al. 201)^61^	https://fragpipe.nesvilab.org/
rMATS-long v2.0.1	Shen et al, 2014^62^	https://github.com/Xinglab/rMATS-long
ESPRESSO v1.3.2	Gao et al. 2023^27^	https://github.com/Xinglab/espresso
SQANTI3 v5.2.1	Pardo-Palacios, et al, 2024.^32^	https://github.com/conesalab/SQANTI3
IsoVis (commit id: d6ebb3e387457ecf5da62c42bb6a2c0b03641ee6)	Wan et al. 2024, ^54^	https://github.com/ClarkLaboratory/IsoVis/
gffcompare v0.12.6	Pertea and Pertea 2020. ^63^	https://github.com/gpertea/gffcompare/
