# Supplementary material for: Long read sequencing reveals novel isoforms and spliceosome-mutant-enriched transcripts in AML and MDS
Source: bioRxiv. 2026 May 27:2026.05.20.726635. Preprint. [Version 2] doi: 10.64898/2026.05.20.726635 (PMC13228619; doi:10.64898/2026.05.20.726635)
Supplement: Supplementary file 1 [file media-10.pdf]

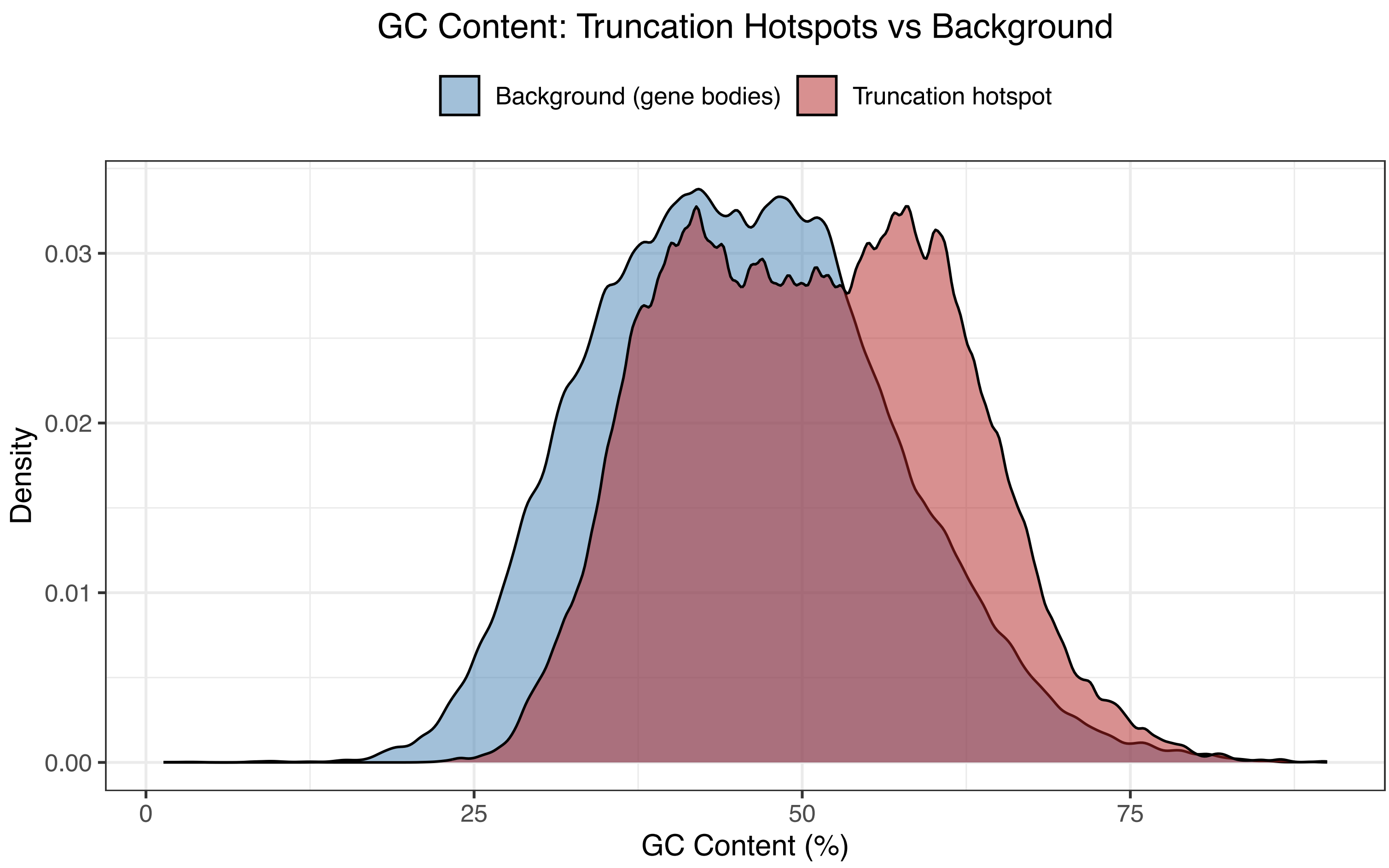

Figure S1: Truncation hotspots have significantly higher GC content than randomly sampled sequences from gene bodies with matched length distributions (Wilcoxon rank sum $p < 2.0e-16$)

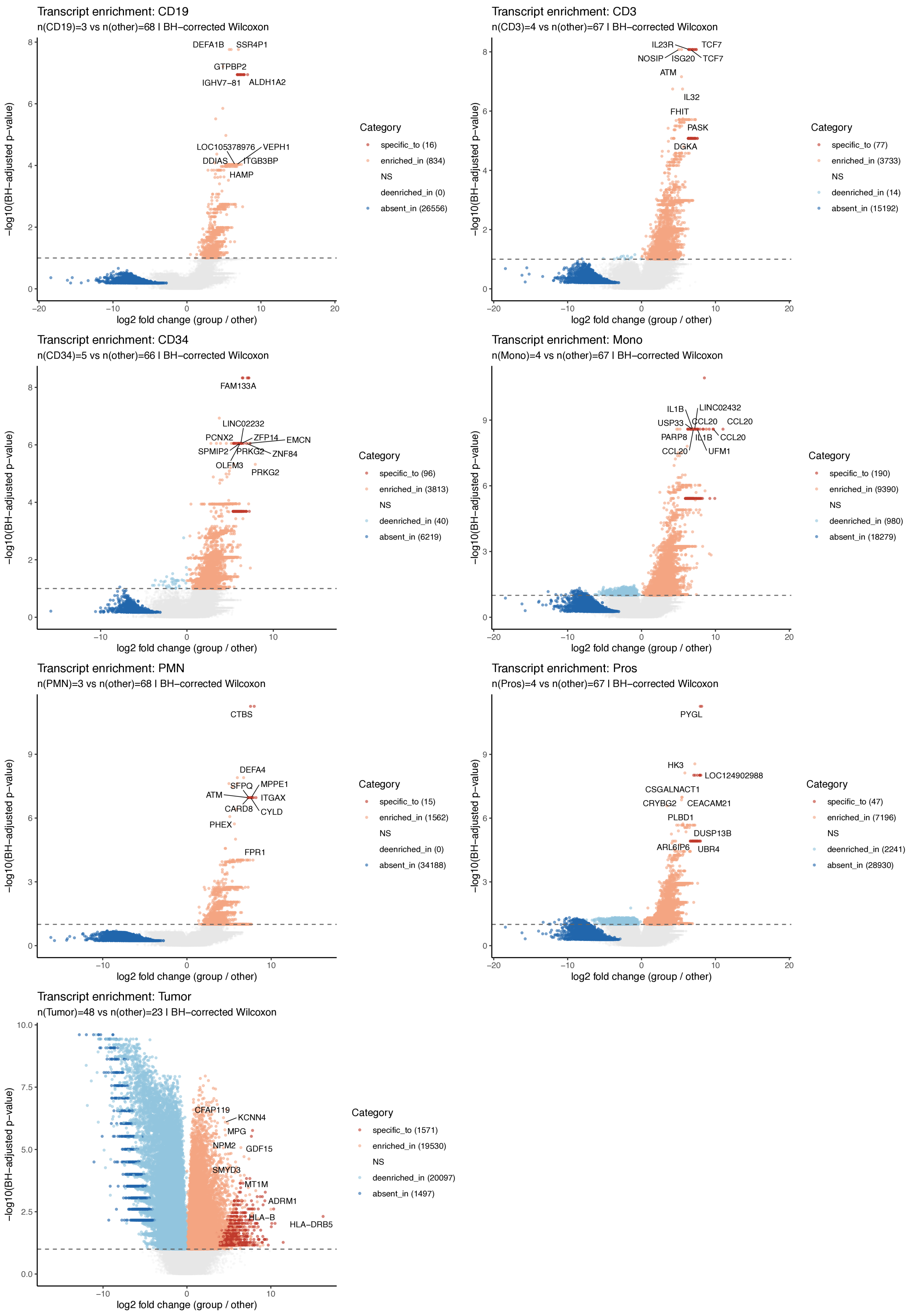

Figure S3: Comparisons between specific populations and all other samples, identifying isoforms that were specific to, enriched in, deenriched in, or absent in specific groups

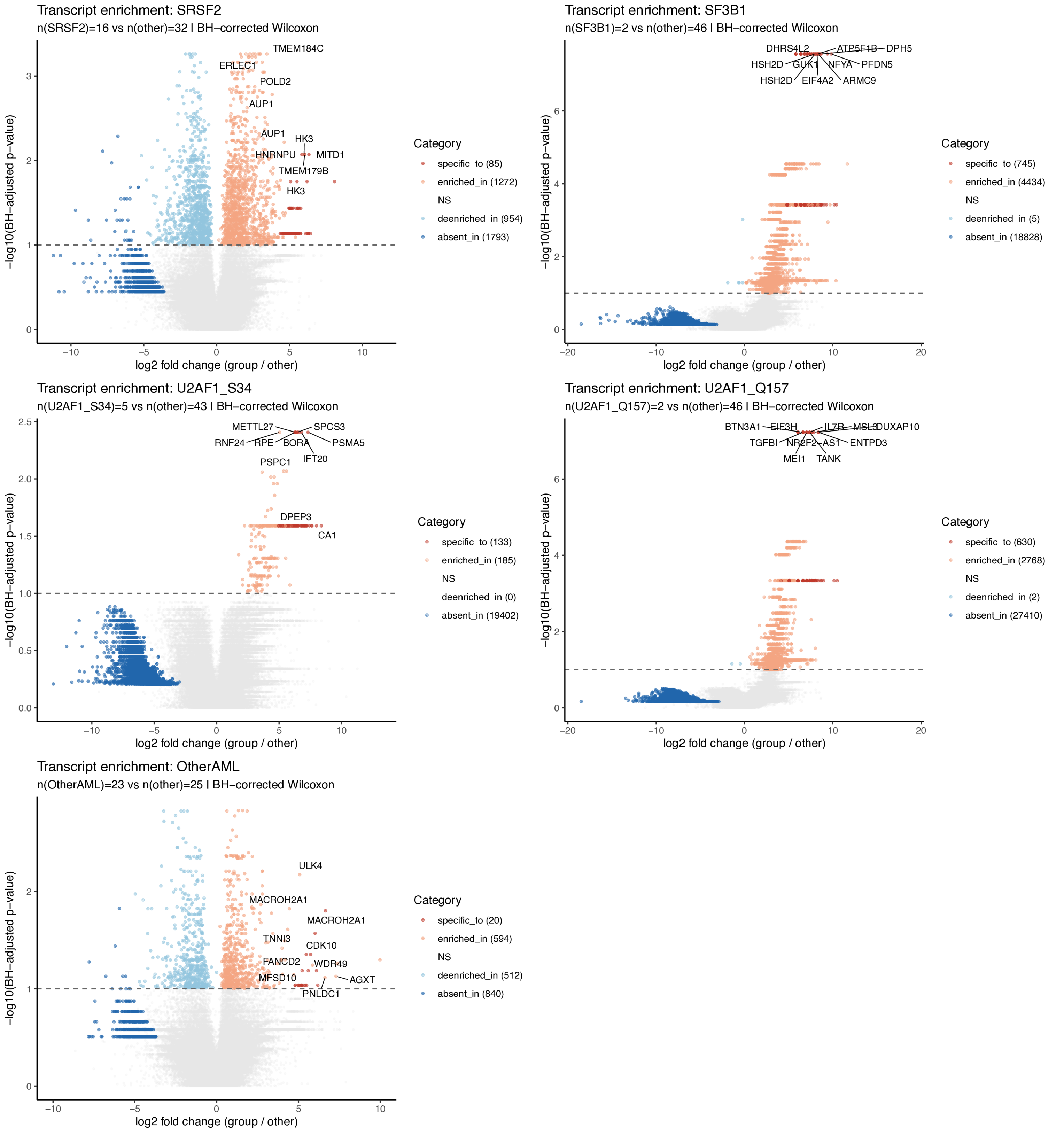

Figure S3: Comparisons between specific tumor groups and other tumor samples, identifying isoforms that were specific to, enriched in, deenriched in, or absent in specific groups
